# Molecular characterization of *Schellackia* parasites in an urban population of sand lizards (*Lacerta agilis*) from Berlin, Germany

**DOI:** 10.1007/s00436-023-07856-w

**Published:** 2023-05-24

**Authors:** Ylva Veith, Anna Luiza Wende, Kai Matuschewski, Juliane Schaer, Katja Müller, Brigitte Bannert

**Affiliations:** Department of Molecular Parasitology, Institute of Biology, Humboldt University, Berlin, Germany

**Keywords:** Apicomplexa, Hemoparasites, *Schellackia* sp*.*, European lizards, *Lacerta agilis*, Urban habitat

## Abstract

Lizards are hosts of several taxa of unicellular parasites of the phylum Apicomplexa, including *Karyolysus, Schellackia, Lankesterella,* and *Hepatozoon*. Parasite prevalence and the impact of infections on lizard biology remain largely unexplored. In this study, blood parasite infections were investigated in sand lizards (*Lacerta agilis*) from Berlin, Germany*.* Eighty-three individuals were investigated, and the detected blood parasites were identified as *Schellackia* sp*.* The combination of microscopic and molecular screening revealed a prevalence of 14.5%. Parasitemia values were low and most infections were subpatent. Phylogenetic analysis recovered a close relationship of the *Schellackia* parasites of this study with *Schellackia* sp*.* parasites of different *Lacerta* and *Podarcis* lizard species from Spain. Monitoring of *Schellackia* parasite infections in free-ranging lizards contributes to a better understanding of the distribution, diversity, and phylogenetic relationships of the neglected parasite taxon.

**Supplementary Information:**

The online version contains supplementary material available at 10.1007/s00436-023-07856-w.

## Introduction

Extant squamates comprise over 10,900 species of lizards, snakes, and amphisbaenians and are the most diverse group of terrestrial vertebrates (Pyron et al. 2013). Hemoparasite infections in lacertid lizards of the Western Palaearctic comprise a variety of parasites such as *Karyolysus* spp., *Lankesterella* spp., *Hepatozoon* spp., and *Schellackia* spp. (Telford 2009; Megía-Palma et al. 2014; Drechsler et al. 2021). The distinct and complex life cycles typically include an invertebrate and a squamate host, and the erythrocytic phase differs between genera. Large parts of the parasites’ life cycle, including asexual replication, gametocyte conversion, zygote formation, and oocyst development in the mucosal epithelium of the duodenum are confined to the vertebrate host (Telford 2009; Megía-Palma et al. 2023). Blood infection occurs after sporozoite release, and infections of both erythrocytes and leucocytes have been described (Bristovetzky and Paperna 1990). In *Schellackia* spp. infections, erythrocyte-infecting sporozoites present the final life cycle step in the lizard host and represent the transmission stages to hematophagous arthropods, which play only a mechanical role as paratenic hosts (Telford 2009). *Schellackia* sporozoites have a circular to oval form and appear white with a notable chromatin stain at the periphery. Another characteristic trait in European species is the presence of one refractile body that stains faintly bluish (Megía-Palma et al. 2014).

Eleven species of *Schellackia* parasites have been described from lizard hosts (Zechmeisterová et al. 2019), and the phylogenetic relationships between the different *Schellackia* species are largely unknown (Megía-Palma et al. 2018). The known distribution of *Schellackia* parasites of lizards comprises Southern and Central Europe, including Spain, Portugal, and Slovakia (Megía-Palma et al. 2013, 2014, 2018; Zechmeisterová et al. 2019; Maia et al. 2014; Kočíková et al. 2018).

Blood infections with *Schellackia* sp. parasites are generally considered non-pathogenic (Telford 2009). However, considerable health impacts on squamate physiology and behavior of infections with other apicomplexan parasites, such as *Plasmodium*, has been documented (Schall et al. 1982). Thus, potential impacts of *Schellackia* infections cannot be excluded as previous studies found complex relationships between different parasites, body condition, and coloration of male lizards (Megía-Palma et al. 2021, 2022).

The sand lizard (*Lacerta agilis,* LINNAEUS 1758) is a medium-sized lizard of the family Lacertidae, with a body length of 70–175 mm (Arnold et al. 2007). Lizards of the family Lacertidae are the most diverse and ubiquitous squamates in the Western Palearctic region and contain about 340 species and present the predominant lizards in Europe (Arnold et al. 2007; Garcia-Porta et al. 2019). Sand lizards inhabit large parts of Europe and Northwestern Asia. Nine subspecies of *L. agilis* are approved, and they are distributed from the south of England, Southern Scandinavia, most of Europe, and eastwards to the Siberian Lake Baikal (Bischoff 1988; Andres et al. 2014). In Germany, *L. agilis* is one of six lizard species and categorized in the red list of endangered lizard species according to the Federal Conservation Office (Blanke et al. 2020). The lizard populations of *L. agilis* suffer from habitat destructions and fragmentation due to land use and construction projects. It remains unknown whether pathogens contribute to the population decline in addition to habitat destruction. Protective measures are being carried out to avoid a further decrease of the lizard populations, for example, by relocating the lizards to an appropriate surrogate habitat before constructions start. In the context of such a relocation project in Berlin, we investigated the blood parasite infections of lizards in their original habitat. The identification of blood parasite infections in wild lizard populations using minimally invasive blood sampling contributes to a better understanding of the prevalence and distribution of hemoparasites of lizards.

## Materials and methods

### Animal handling and blood collection

In the context of a relocation project in Berlin Lichterfelde Süd in the year 2021, lizards were caught in their original habitat, which was in part a former military training area of the US Army and were brought to a nearby compensation habitat.

Animal procedures were performed in accordance with the "German Tierschutzgesetz in der Fassung vom 18. Mai 2006" (BGBl.I S.1207) which implements the directive 2010/6 3/EU of the European Union. The protocol was approved by the ethics committee of the Berlin state authority (“Landesamt für Gesundheit und Soziales Berlin,” permit number O0163/20). Reptile fences were set up over the area to capture the lizards and relocate them to their new habitats. Along the reptile fences, bucket traps, with a depth of 20 cm, were placed every 15 m. Every trap was completely placed into the ground and was labelled with an individual number. To secure the captured lizards, holes were put into the bottom of the trap to ensure the drain of water and bucket lids were placed using three sticks attached to the bucket rim and leaving a gap in between. The fences and traps were monitored daily.

**Fig. 1:**
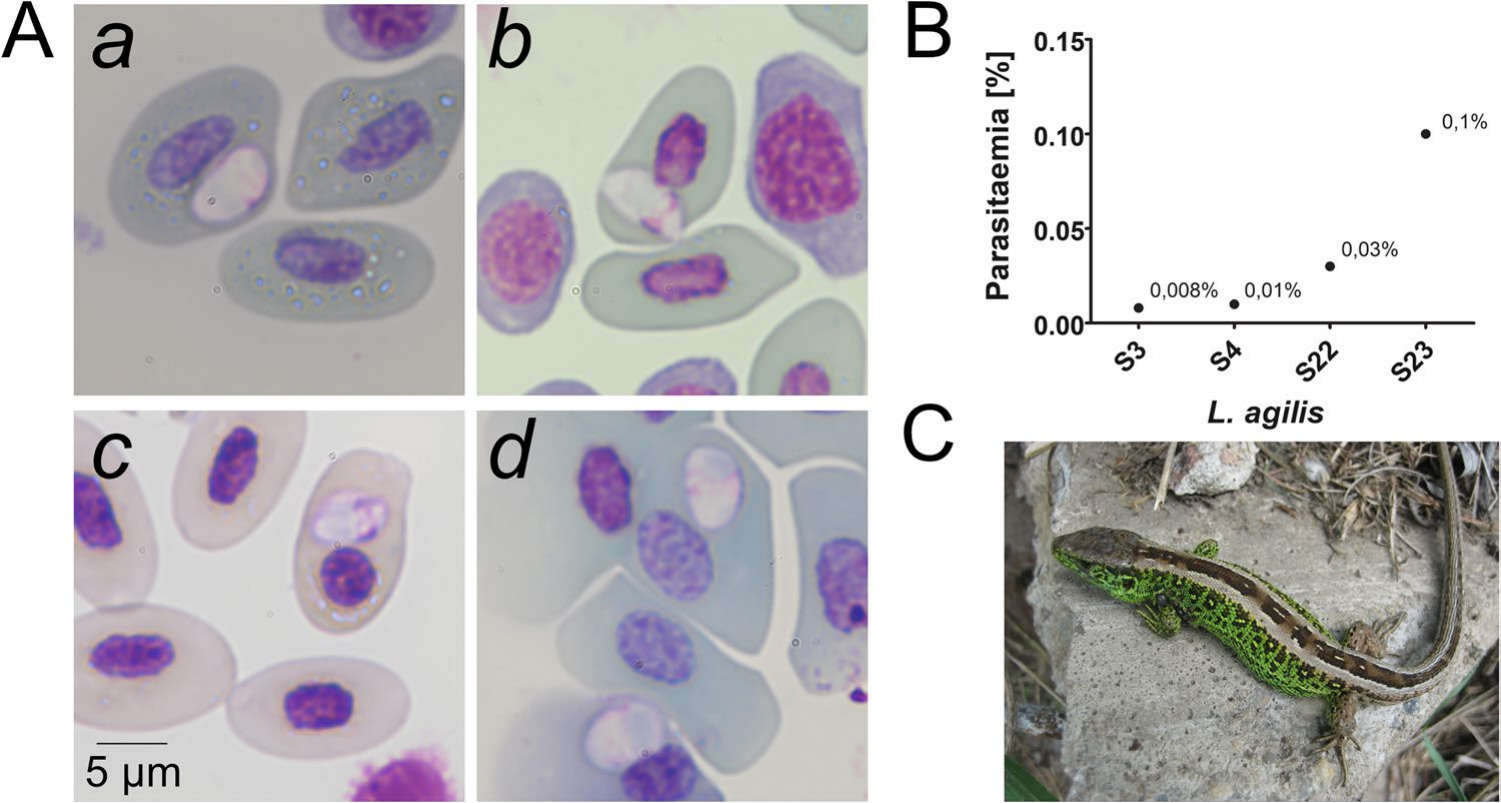
*Schellackia* sp. infections in urban *Lacerta agilis. ***A** Representative micrographs (Giemsa-stain) of *Schellackia* sp. parasites of *L. agilis* from Berlin (**a**, sample S3; **b**, sample S4; **c**, sample S22; **d**, sample 23). Sporozoites were limited to erythrocytes. A single refractile body (that stains faintly bluish) is visible in each parasite. Size bars = 5 μm. **B** Parasitemia values in the four patent *Schellackia* sp. infections in *L. agilis.* **C** Photograph of an adult male of *L. agilis* with an engorged larvae of a tick attached (front left limb), which are potential passive vectors of *Schellackia* parasites

Blood samples were collected from 83 *L. agilis* (Fig. 1C) individuals captured in the region of Lichterfelde Süd (52°24′37.5″ N 13°18′41.4″ E), Berlin, Germany, from May 20 until August 19, 2021. The ventral tail vein of the lizards was carefully punctured using a 23-gauge needle to obtain 3 μl blood for sampling. Two blood smears and blood dots on Whatman® filter paper were collected for each individual. After sample collection each lizard was monitored for several minutes to ensure that the lizard was not impaired before being released in the compensation habitat.

### Microscopy

Thin blood smears on microscopic slides were fixed in 100% methanol and stained with a 5% Giemsa solution for 30 min. For microscopic examination, the stained blood smears were screened in at least 100 microscopic fields (about 10,000 erythrocytes per slide) using a light microscope (Zeiss Axioplan, Carl Zeiss, Oberkochen, Germany) with a magnification of 1,000. Images of parasites were taken with a Canon EOS 700D. Length and width of the sporozoite parasite stages were measured, and the images were edited using Adobe Photoshop.

### Molecular detection

DNA was extracted from all samples using the DNeasy Blood and Tissue Kit (QIAGEN GmbH, Hilden, Germany) according to the manufacturer’s instructions. PCRs targeting approximately 1,000 bp of the 18S ribosomal RNA (rRNA) of *Schellackia* sp. were carried out for all samples using DreamTaq polymerase (Thermo Fisher) and the published primers Hep600F1 and Hep1600R (Megía-Palma et al. 2013), with the temperature protocol 95° C for 10 min, followed by 40 cycles of 95° C for 30 s, 58° C for 30 s, 72° C for 120 s, and a final extension at 72 °C for 10 min. All positive samples were Sanger sequenced using the amplification primers.

### Phylogenetic analysis

**Fig. 2:**
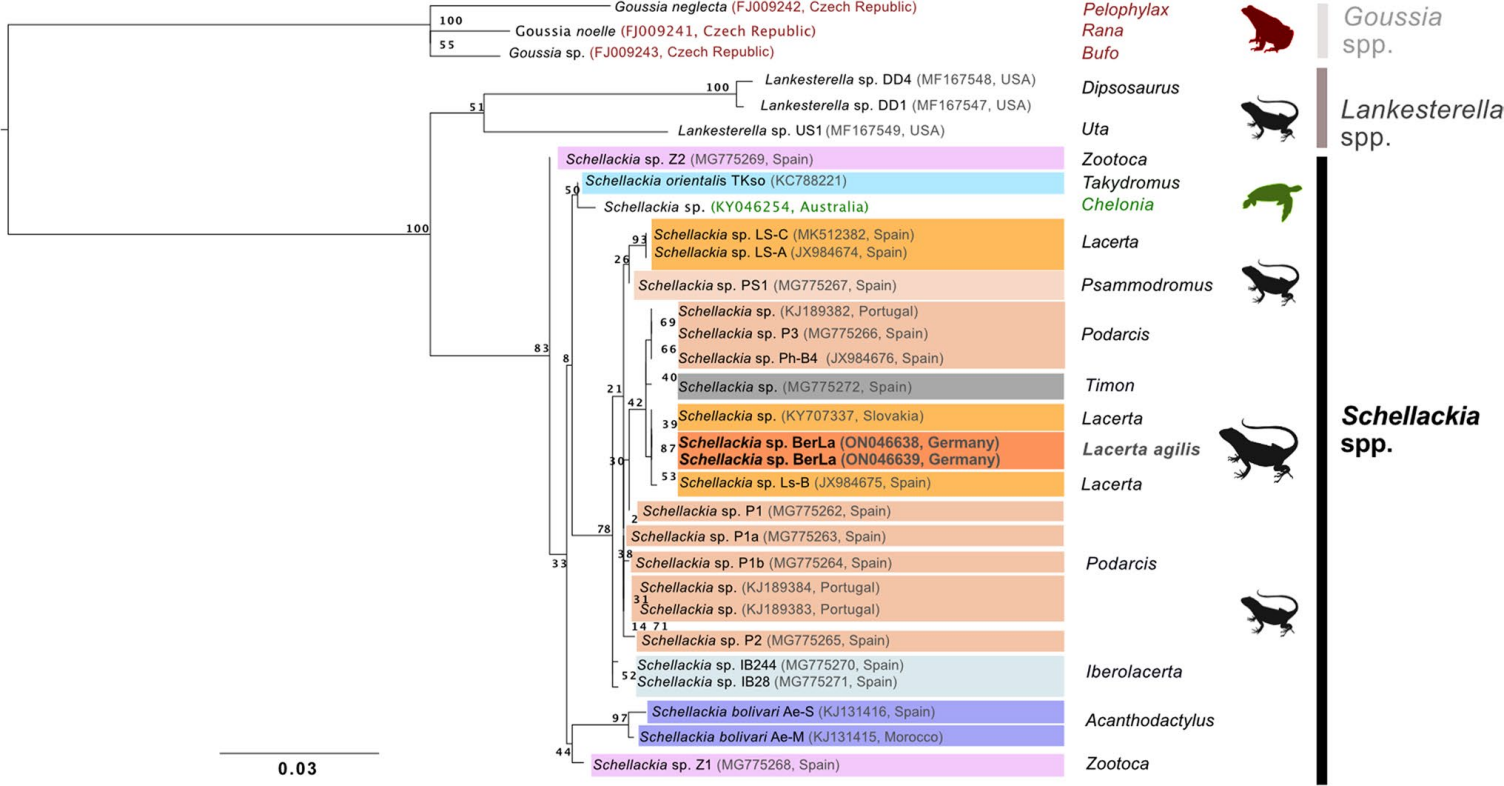
Maximum likelihood analysis of the 18S rRNA of Schellackia parasites and selected closely related taxa. Two representative sequences of this study (BerLa) are highlighted in bold and dark orange. The two sequences of the BerLa haplotype group with Schellackia spp. haplotypes of three different host genera: Lacerta, highlighted in light orange Podarcis, highlighted in brown; and Timon, highlighted in dark brown. Non-squamate-hosts are labeled with genus/species names (Bufo, common toad; Chelonia mydas, green sea turtle; Rana dalmatina, agile frog; Pelophylax esculentus, edible frog)

Nucleotide sequences were edited manually and were compared to reference sequences available in NCBI GenBank using the BLASTn algorithm (http://blast.ncbi.nlm.nih.gov/Blast.cgi) (Tab. S2). Nucleotide sequences were quality checked, and ambiguous base calls or missing data were coded with N´s or the corresponding ambiguity code. Sequences were aligned using the MAFFT algorithm (Katoh et al. 2002; Katoh and Standley 2013). Sequence divergence was calculated, and haplotype diversity was assessed in Geneious Prime. Reference sequences including sequences from previous molecular studies of *Schellackia* spp. and representatives of hemococcidian taxa were retrieved from GenBank and added to the alignments of the sequences of the present study. All GenBank accession numbers are listed in Table S2. Phylogenetic analysis of the 18S rRNA data set was carried out with ModelTest-NG to test different DNA substitution models (Darriba et al. 2019). The alignment of the partial 18S rRNA of a total of 1,019 nucleotides (nt) comprised 43 sequences, including two sequences of this study. Maximum likelihood (ML) analysis using the substitution model TIM3 + I (proportion of invariant sites) + Gamma (rate heterogeneity) was conducted in raxmlGUI version 2.0.6 (Edler et al. 2021). Nodal support was evaluated using 1000 thorough bootstrap and consensus. Sequences of *Goussia* spp., parasitic protists that infect fish and amphibians, were used as outgroups and to root the phylogenetic tree (Fig. 2). Phylogenetic trees were visualized with FigTree v1.4.4 (http://tree.bio.ed.ac.uk/software/figtree/). To highlight the tree, affinity photo was used (https://affinity.serif.com/de/photo/).

## Results and discussion

In this study, a total of 83 individuals of *L. agilis* were investigated. The majority, 46%, were females, 29% were males, and 25% subadult lizards (Tab. S3). Microscopic and molecular analysis of the blood samples revealed infections with *Schellackia* sp. parasites in *L. agilis*. The investigation of *Schellackia* sp. infections by microscopy discovered four infected individuals (Fig. 1Aa–d). However, the molecular screening detected *Schellackia* sp. infections in twelve out of 83 lizards (prevalence = 14.5%). Thus, eight out of twelve infections were rated as subpatent infections. This finding underscores the importance of systematic molecular diagnostic screening to include subpatent infections in cross-sectional and longitudinal studies. Higher prevalences of *Schellackia* sp. infections in *Lacerta schreiberi* were reported in Spain, i.e., 29.5% (Megía-Palma et al. 2013) and 36.7%, (Megía-Palma et al. 2018), respectively. However, a very low prevalence (1 out of 49) was reported from a screen for *Schellackia* sp. infections in *L. viridis* (Kočíková et al. 2018). In studies of other lizard species, the mean prevalence of *Schellackia* sp. infections varied considerably, i.e., in *Zootoca vivipara* 4.8%, *Timon lepidus* 38%, and *Podarcis vaucheri* 90% (Megía-Palma et al. 2018).

In *L. agilis* from Berlin, *Schellackia* sporozoites were exclusively detected in erythrocytes. Additionally, no extracellular parasites were noted. The parasites tended to be located close to the nucleus of the lizard’s erythrocyte (Fig. 1A). However, there was no indication for a displacement of the erythrocytic nucleus, and instead the parasites take space by bulging the erythrocyte (Fig. 1A(a,c)). The mean sizes of the *Schellackia* sp. sporozoites (mean length and widths in µm ± SD) of the infected lizards from Berlin are 5.9 (±0.7) μm in length and 3.7 (±0.7) μm in width (Tab. S4). The sizes of the sporozoites correspond to the reported sporozoite sizes of *Schellackia* sp. infecting *L. schreiberi* and *Schellackia* sp. infecting *Podarcis hispanicus* from Spain, which display 5.3–5.9 µm in length and 3–3.4 µm in width (Tab. S4) (Megía-Palma et al. 2013).

The parasitemia values of *Schellackia* sp. infections in *L. agilis* from Berlin range from 0.008 to 0.1%, corresponding to a mean of 0.034% (Fig. 1B). The parasitemia of *Schellackia* sp. in *L. agilis* of this study is in line with previous studies, which reported similar values of parasitemia in *L. schreiberi*, i.e., for *L. schreiberi* from the Iberian Peninsula close to the Portuguese–Spanish border a mean parasitemia of 0.04% of *Schellackia* sp. (Zechmeisterová et al. 2019), and *L. schreiberi* lizards from Segovia (Spain) featured a mean parasitemia of 0.09% (Megía-Palma et al. 2013).

Nucleotide sequences (18S rRNA) with high quality were obtained from eleven samples and used for subsequent molecular analysis. The 18S rRNA sequences of the parasites of this study were identical and thus represent one haplotype of *Schellackia* sp. (BerLa). Sequences of two representative samples (GenBank accession numbers ON046638 and ON046639) were included in the subsequent phylogenetic analysis to explore the phylogenetic relationships of the detected *Schellackia* parasites among the larger group of *Schellackia* parasites and closely related taxa. The maximum likelihood analysis (18S rRNA) recovered two clades with high support (bootstrap value of 100). One clade comprises sequences of *Lankesterella* spp. (dark gray bar), parasitic alveolates that infect amphibians, squamates, and birds. The second clade comprises all sequences of *Schellackia* spp. (black bar), including the sequences of this study (Fig. 2, highlighted in dark orange; other *Schellackia* sequences of hosts of the genus *Lacerta* are highlighted in light orange). The clade further includes sequences of *Schellackia* spp. found in several lizard species of different genera (*Podarcis*, brown; *Psammodromus*, light brown; *Timon*, dark gray; and *Iberolacerta* light blue). The more distant related *Schellackia*-sequences originate from the related species *Schellackia orientalis* and *Schellackia bolivari.* The *Schellackia* sequences of this study feature a close relationship with *Schellackia* sp. isolates from the European green lizard from Slovakia (*Lacerta viridis*) and from the Iberian emerald lizard from Spain (*Lacerta schreiberi*) (Fig. 2).

The overlap of only 256 bp of the *Schellackia* sp. haplotype infecting *L. viridis* from Slovakia (Kočíková et al. 2018) with the *Schellackia* sp. haplotype BerLa is very short. Thus, more data from the Slovakian parasite is needed to confirm this finding. The sequences of *Schellackia* parasites of the present study feature highest identity with the *Schellackia* sp. haplotype Ls-B of *L. schreiberi* from Spain (sequence overlap of 951 bp with one base difference) (Megía-Palma et al. 2013). Thus, according to the 18S partial sequences, these two host species seem to be infected by two different haplotypes of the same *Schellackia* species. However, the geographic ranges of the two lizards do not overlap, and evidence for a high host specificity of various *Schellackia* sp. haplotypes has been provided. *L. agilis* is present in the Iberian Peninsula in small northern populations and future studies may provide interesting information regarding host specificity (Megía-Palma et al. 2018). Further research is warranted to explore the phylogenetic relationships of the *Schellackia* parasites of *L. agilis*, *L. viridis* and *L. schreiberi* in more detail. Molecular genotyping of lizard infecting *Schellackia* parasites is currently limited to 18S rRNA sequences. Further studies on additional mitochondrial and nuclear genes would greatly improve the resolution of the phylogenetic relationships of the *Schellackia* parasites. Moreover, studies in free-ranging lizards in Europe are needed to understand the occurrence, distribution, diversity, and evolution of *Schellackia* parasites and their potential impact on the lizard populations.

## Supplementary Information

Below is the link to the electronic supplementary material.

Supplementary file1 (PDF 348 KB)

## Data Availability

All sequence data have been deposited to GenBank (https://www.ncbi.nlm.nih.gov/genbank/) and are available under the respective accession numbers.
