# Supplementary material for: Molecular characterization of Schellackia parasites in an urban population of sand lizards (Lacerta agilis) from Berlin, Germany
Source: Parasitol Res. 2023 May 24;122(8):1759–64. doi: 10.1007/s00436-023-07856-w (PMC10348933; doi:10.1007/s00436-023-07856-w)
Supplement: Supplementary file 1 — Supplementary file1 (PDF 348 KB) [file 436_2023_7856_MOESM1_ESM.pdf]

SUPPLEMENTARY INFORMATION

Molecular characterization of *Schellackia* parasites in an urban population of sand lizards (*Lacerta agilis*) from Berlin, Germany

Ylva Veith, Anna Luiza Wende, Kai Matuschewski, Juliane Schaer, Katja Müller, Brigitte Bannert

Content:

- **Supplemental Tables 1-4**
- **Supplemental References**

Veith et al., Supplemental Table S1

Table S1: Oligonucleotides for amplification of 18S rRNA of *Schellackia* parasites

Primer	Sequence 5'- 3'	Author
Hep600F1	TCG TAG TTG GAT TTC TGT CG	MEGÍA-PALMA et al., 2013
Hep1600R	AAA GGG CAG GGA CGT AAT CGG	MEGÍA-PALMA et al., 2013

Veith et al., Supplemental Table S2

Table S2: Gene accession numbers of sequences included in phylogenetic analysis

**Type**	**GenBank Nr**	**Host species**	**Author**	**Locality**
Schellackia sp. BerLa	**ON046638**	*Lacerta agilis*	Current study	Germany: Berlin
Schellackia sp. BerLa	**ON046639**	*Lacerta agilis*	Current study	Germany: Berlin
Schellackia sp.	KY046254	*Chelonia mydas*	DE GOUVEA PEDROSO et al., 2020	Australia
Schellackia sp. Ls-C	MK512382	*Ixodes ricinus* fed on *Lacerta schreiberi*	ZECHMEISTEROVÁ et al., 2019	Spain
Schellackia sp.	KY707337	*Lacerta viridis*	Kočíková et al., 2018	Slovakia: Zadiel
Schellackia sp. P1	MG775262	*Podarcis guadarramae*	MEGÍA-PALMA et al., 2018	Spain: Madrid, Segovia, Cuenca, Huesca
Schellackia sp. P1a	MG775263	*Podarcis guadarramae*	MEGÍA-PALMA et al., 2018	Spain: Guadarrama Mountains
Schellackia sp. P1b	MG775264	*Podarcis muralis*	MEGÍA-PALMA et al., 2018	Spain: Huesca
Schellackia sp. P2	MG775265	*Podarcis muralis*	MEGÍA-PALMA et al., 2018	Spain: Madrid
Schellackia sp. P3	MG775266	*Podarcis muralis*	MEGÍA-PALMA et al., 2018	Spain: Leon, Madrid, Segovia, Chafarinas Islands, Toledo, Lerida
Schellackia sp. PS1	MG775267	*Psammodromus algirus*	MEGÍA-PALMA et al., 2018	Spain: Segovia, Madrid, Toledo, Valencia, and Huelva
Schellackia sp. Z1	MG775268	*Zootoca vivipara*	MEGÍA-PALMA et al., 2018	Spain: Huesca, Irun
Schellackia sp. Z2	MG775269	*Zootoca vivipara*	MEGÍA-PALMA et al., 2018	Spain: Huesca
Schellackia sp. IB244	MG775270	*Iberolacerta cyreni*	MEGÍA-PALMA et al., 2018	Spain: Madrid
Schellackia sp. IB28	MG775271	*Iberolacerta monticola*	MEGÍA-PALMA et al., 2018	Spain: Asturias, Leon, Pyrenees Mountains
Schellackia sp.	KJ189384	*Podarcis hispanicus*	MAIA et al., 2014	Portugal: Geres

Schellackia sp.	KJ189383	*Podarcis bocagei*	MAIA et al., 2014	Portugal: Geres
Schellackia sp.	KJ189382	*Podarcis bocagei*	MAIA et al., 2014	Portugal: Geres
Schellackia bolivari Ae-M	KJ131415	*Acanthodactylus erythrurus*	MEGÍA-PALMA et al., 2014	Morocco
Schellackia bolivari Ae-S	KJ131416	*Acanthodactylus erythrurus*	MEGÍA-PALMA et al., 2014	Spain: Madrid
Schellackia sp. 1 JM 2013 Ph-B4	JX984676	*Podarcis hispanicus*	MEGÍA-PALMA et al., 2013	Spain
Schellackia sp.	MG775272	*Timon lepidus*	MEGÍA-PALMA et al., 2013	Spain: Segovia
Schellackia sp. LS-A	JX984674	*Lacerta schreiberi*	MEGÍA-PALMA et al., 2013	Spain: Segovia
Schellackia sp. LS-B	JX984675	*Lacerta schreiberi*	MEGÍA-PALMA et al., 2013	Spain: Segovia
Schellackia orientalis TKso	KC788221	*Takydromus sexlineatus*	MEGÍA-PALMA et al., 2013	-
Lankesterella sp. DD4	MF167548	*Dipsosaurus dorsalis*	MEGÍA-PALMA et al., 2017	USA
Lankesterella sp. DD1	MF167547	*Dipsosaurus dorsalis*	MEGÍA-PALMA et al., 2017	USA
Lankesterella sp. US1	MF167549	*Uta stansburiana*	MEGÍA-PALMA et al., 2017	USA
Goussia sp.	FJ009243	*Bufo bufo*	JIRKŮ et al., 2009	Czech Republic
Goussia noelleri	FJ009241	*Rana dalmatina*	JIRKŮ et al., 2009	Czech Republic
Goussia neglecta	FJ009242	*Pelophylax esculentus*	JIRKŮ et al., 2009	Czech Republic

Veith et al., Supplemental Table S3

Table S3: *Lacerta agilis* captures and *Schellackia* sp. PCR screening (18S rRNA) results

**Results**	**Females**	**Males**	**Subadults**	**Total**
negative	34	18	18	70
positive	4	6	3	13
Total	38	24	21	83

Veith et al., Supplemental Table S4

Table S4: Comparison of sporozoite sizes of *Schellackia* sp. of *L. agilis* of this study with *Schellackia* sporozoites of *L. schreiberi* and *P. hispanicus*

			**Sporozoite sizes**				
			**Length (µm)**		**Width (µm)**		
**Sample**	**Host**	**N**	**Mean (± SD)**	**Range**	**Mean (± SD)**	**Range**	**Locality**
BerLa*	*Lacerta agilis*	24	5.9 (± 0.7)	4.2 - 7.0	3.7 (± 0.7)	2.4 - 5.0	Germany (Berlin)
Ls-A**	*Lacerta schreiberi*	64	5.3 (± 0.6)	4.0 - 6.8	3.4 (± 0.5)	2.3 - 4.7	Spain: Segovia
Ls-B**	*Lacerta schreiberi*	24	5.6 (± 0.8)	4.7 - 8.5	3.3 (± 0.6)	2.2 - 4.4	Spain: Segovia
1 JM 2013 Ph-B4**	*Podarcis hispanicus*	5	5.9 (± 0.7)	5.1 - 6.8	3.0 (± 0.4)	2.6 - 3.3	Spain: Segovia

*source: this study; **source: Megia Palma et al. 2013

Supplemental References

de Gouvea Pedroso SB, Phalen DN, Terkildsen M, Blyde D, March DT, Gordon AN, Chapman PA, Mills PC, Owen H, Gillett A, Lloyd HB, Ross GA, Hall J, Scott J, Ariel E, Yang R, Rose KA (2020) Coccidiosis in green turtles (*Chelonia mydas*) in Australia: Pathogenesis, spatial and temporal distribution, and climate-related determinants of disease outbreaks. J Wildl Dis 56:359-371. https://doi.org/10.7589/2019-05-115

Jirku M, Jirku M, Obornik M, Lukes J, Modry D (2009) *Goussia* Labbé, 1896 (Apicomplexa, Eimeriorina) in Amphibia: Diversity, biology, molecular phylogeny and comments on the status of the genus. Protist 160:123-136. https://doi.org/10.1016/j.protis.2008.08.003

Kočíková B, Majláth I, Majláthová V (2018) The occurrence of protozoan parasites (*Schellackia* sp. Reichenow, 1919, *Tritrichomonas* sp. Kofoid, 1920, and *Proteromonas* sp. Kunstler, 1883) in Lacertid lizards from selected localities in Europe. Comp Parasitol 85:48-57. https://doi.org/10.1654/1525-2647-85.1.48

Maia JP, Harris DJ, Carranza S, Gomez-Diaz E (2014) A comparison of multiple methods for estimating parasitemia of Hemogregarine Hemoparasites (Apicomplexa: Adeleorina) and its application for studying infection in natural populations. PLoS One 9:e95010. https://doi.org/10.1371/journal.pone.0095010

Megía-Palma R, Martínez J, Merino S (2013) Phylogenetic analysis based on 18S rRNA gene sequences of *Schellackia* parasites (Apicomplexa: Lankesterellidae) reveals their close relationship to the genus *Eimeria*. Parasitol 140:1149–1157. https://doi.org/10.1017/S0031182013000553

Megía-Palma R, Martínez J, Merino S (2014) Molecular characterization of haemococcidia genus *Schellackia* (Apicomplexa) reveals the polyphyletic origin of the family Lankesterellidae. Zool Scripta 43:304–312. https://doi.org/10.1111/zsc.12050

Megía-Palma R, Martínez J, Paranjpe D, D'Amico V, Aguilar R, Palacios MG, Cooper R, Ferri-Yanez F, Merino S (2017) Phylogenetic analyses reveal that *Schellackia* parasites (Apicomplexa) detected in American lizards are closely related to the genus *Lankesterella*: is the range of *Schellackia* restricted to the Old World? Parasite Vect 10:470. https://doi.org/10.1186/s13071-017-2405-0

Megía-Palma R, Martínez J, Cuervo JJ, Belliure J, Jiménez-Robles O, Gomes V, Cabido C, Pausas JG, Fitze PS, Martín J, Merino S (2018) Molecular evidence for host-parasite co-speciation between lizards and *Schellackia* parasites. Int J Parasitol 48:709-718. https://doi.org/10.1016/j.ijpara.2018.03.003

Zechmeisterová, K, De Bellocq JG, Široký P (2019) Diversity of *Karyolysus* and *Schellackia* from the Iberian lizard *Lacerta schreiberi* with sequence data from engorged ticks. Parasitol 146:1690-1698. https://doi.org/10.1017/S0031182019001112
